# Supplementary material for: Drosophila Trus, the orthologue of mammalian PDCD2L, is required for proper cell proliferation, larval developmental timing, and oogenesis
Source: bioRxiv. 2024 Oct 26:2024.10.24.620039. Preprint. [Version 1] doi: 10.1101/2024.10.24.620039 (PMC11527112; doi:10.1101/2024.10.24.620039)
Supplement: 1 [file NIHPP2024.10.24.620039v1-supplement-1.pdf]

bioRxiv preprint doi: https://doi.org/10.1101/2024.10.24.620039; this version posted October 26, 2024. The copyright holder for this preprint (which was not certified by peer review) is the author/funder, who has granted bioRxiv a license to display the preprint in perpetuity. It is made available under aCC-BY 4.0 International license.

Supplementary Table 1. Drosophila lines that are used in this study.

Category	Name used in this paper	Genotype	Source	Stock number or publication
Control	w^{1118}	*w[1118]*	BDSC	BDSC_5905
CRISPR/Cas9	vas-Cas9	*y[1] M{RFP[3xP3.PB] GFP[E.3xP3] =vas-Cas9}ZH-2A w[1118]/FM7c*	BDSC	BDSC_51323
Balancer	TM6BSbTbHuYFP	*w[*]; Bl/CyOGFP; TM2/TM6BP[Dfd-GMR-nvYPF]SbTb*	lab stock	Le et al. 2006
PhiC31 source	dPhiC3	*y[1] M{RFP[3xP3.PB] GFP[E.3xP3] =vas-int.Dm}ZH-2A w[*]*	BestGene	BDSC_40161

bioRxiv preprint doi: https://doi.org/10.1101/2024.10.24.620039; this version posted October 26, 2024. The copyright holder for this preprint (which was not certified by peer review) is the author/funder, who has granted bioRxiv a license to display the preprint in perpetuity. It is made available under aCC-BY 4.0 International license.

attP line	VK37	*y[1] w[1118]; PBac{y[+]-attP-3B}VK00037*	BestGene	BDSC_9752
RNAi	UAS-Trus RNAi	*w^{1118}; P{GD11610}v22067*	VDRC	v22067
	UAS-DilpRNAi	*P{KK112161}VIE-260B* on 2nd	VDRC	v102604
	UAS-Xrp1RNAi	*P{KK104477}VIE-260B* on 2nd	VDRC	v107860
	UAS-dicer2	*w[1118]; ; P{w[+mC]=UAS-Dcr-2.D}10*	BDSC	BDSC_24651
GAL4 drivers	da-GAL4	*w[*]; ; P{w[+mW.hs]=GAL4-da.G32}UH1*	lab stock	BDSC_55850
	nub-GAL4	*w[*]; P{w[nub.PK]=nub-GAL4.K}2*	BDSC	BDSC_86108
	pdm2-GAL4	*w[1118]; P{y[+t7.7]w[+mC]=GMR11F02-GAL4}attP2*	BDSC	BDSC_49828
	ci-GAL4	*w[1118]; P{ci-GAL4.C}*	Herman Steller	Croker et al. 2006
	en-GAL4	*y[1] w[*]; P{w[+m*]=GAL4}en[GAL4-33]*	BDSC	BDSC_99568
	ci, en-GAL4	combination of ci-GAL4 and en-GAL4 on 2nd	lab stock	
	repo-GAL4	*w[1118]; P{w[+m*]=GAL4}repo/TM3,Sb[1]*	BDSC	BDSC_7415
	spok-GAL4	*w[1118]; ; P{spok-GAL4,mw+}16A3,17A3*	lab stock	Shimell and O'Connor 2023
	e22c-GAL4	*w[*]; P{w[+mW.hs]=en2.4-GAL4}e22c*	BDSC	BDSC_1973
	elav-GAL4	*P{w[+mC]=GAL4-elav.L}2/CyO*	BDSC	BDSC_8765
UAS lines	Dilp8-GFP	*y[1] w[*]; Mi{y[+mDint2]=MIC}Ilp8[MI00727]*	BDSC	BDSC_33079
	UAS-Trus	*w[1118]; P[UAS-Trus,mw+]VK37*	this study	
	UAS-EGFP-Trus	*w[1118]; P[UAS-EGFP-Trus,mw+]VK37*	this study	
	UAS-p35	*w[*]; P{w[+mC]=UAS-p35.H}BH1*	BDSC	BDSC_5072
	mGFP;RedStinger	*P{UAS-mGFP}; ; P{w[+mC]=UAS-RedStinger}*	Jae Park	
trus mutants	$trus^{4-15}$	*w[1118] ; ; trus[4-15]/TM6B P[Dfd-GMR-nvYPF]SbTb*	this study	
	$trus^{35-2}$	w[1118] ; ; trus[35-2]/TM6B P[Dfd-GMR-nvYPF]SbTb	this study	
	$trus^{1}$	*w[1118] ; ; trus[1]/TM6B P[Dfd-GMR-nvYPF]SbTb*	Zucker EMS mutant collection	Koundakjian et al. 2004
	Df trus	*w[1118]; ; Df(3R)BSC847/TM6C, Sb1 cu1*<br>3R: 12,372,864..12,562,460	BDSC	BDSC_27920
Zfrp8 mutants	Zfrp8P	*y[1] w[67c23]; P{lacW}Zfrp8k13705/CyO*	BDSC	BDSC_12199
	Df Zfrp8	*w[1118]; Df(2R)BSC356/SM6a*<br>2R:24,068,239..24,257,904 (189,666 bp)	BDSC	BDSC_24380
	Df(2R)SM206 Zfrp8 null	*Df(2R)SM206 /CyOGFP*	Ruth Steward	Minakhina et al. 2003
	$Zfrp8^{M-1-1}$	*Zfrp8[M-1-1] /CyOGFP*	Ruth Steward	Minakhina et al. 2007

bioRxiv preprint doi: https://doi.org/10.1101/2024.10.24.620039; this version posted October 26, 2024. The copyright holder for this preprint (which was not certified by peer review) is the author/funder, who has granted bioRxiv a license to display the preprint in perpetuity. It is made available under aCC-BY 4.0 International license.

Supplementary Table 2. DNA oligos that are used for production of CRISPR/Cas9 trus mutants.

Name	Sequence	Use
target A sense	5’ CTTC GGAATGGTCACCTCGTGTCT 3’	CRISPR mutant
target A antisense	5’ AAAC AGACACGAGGTGACCATTCC 3’	CRISPR mutant
target B sense	5’ CTTC GGATACGATCCCGCTGTTGG 3’	CRISPR mutant
target B antisense	5’ AAAC CCAACAGCGGGATCGTATCC 3’	CRISPR mutant
Trus1 for	5’ GTACCTAGGATACGAGGATG 3’	sequencing
Trus2 for	5’ GAAAGACTTGAATGAAACCATG 3’	PCR
Trus3 rev	5’ CATGACGGAATGGTCACC 3’	sequencing
Trus1 rev	5’ GTTGCACCAGTCTGCAATC 3’	PCR and sequencing
TrusB for	5’ CAACCGAACAGGCCAAG 3’	sequencing
TrusC for	5’ CAGGAGTACAAGCTGAGAG 3’	sequencing

bioRxiv preprint doi: https://doi.org/10.1101/2024.10.24.620039; this version posted October 26, 2024. The copyright holder for this preprint (which was not certified by peer review) is the author/funder, who has granted bioRxiv a license to display the preprint in perpetuity. It is made available under aCC-BY 4.0 International license.

Fig.S1 *trus*^{1} mutant shows 'Minute' syndrome phenotype. *trus*^{1} mutant (*trus*^{1}*/Dftrus*) larvae delay development, and most of them are pre-pupal lethal. Rare escaper adults show rough/notched eyes and thin/short bristles which resemble the haplo-insufficiency 'Minute' syndrome that is often observed in flies carrying a mutation in one of the genes encoding ribosomal proteins.

Fig.S2 Overexpression of p35, an apoptosis inhibitor, did not rescue the defects in tissue growth and cell proliferation in *trus* mutant larvae. Representative images of brain (top row) and wing discs (bottom row) from *da>UAS-p35* in *trus*$^{4\text{-}15}$*/Dftrus* third instar wandering larvae are shown. In the left column, DNA staining with DAPI (green) is merged with anti-PH3 antibody

bioRxiv preprint doi: https://doi.org/10.1101/2024.10.24.620039; this version posted October 26, 2024. The copyright holder for this preprint (which was not certified by peer review) is the author/funder, who has granted bioRxiv a license to display the preprint in perpetuity. It is made available under aCC-BY 4.0 International license.

staining (magenta). Individual channels are shown in the middle and right columns. Brain, leg discs, and wing discs are smaller than those dissected from wild type (Fig.1D) and their structures are disturbed. Scale bar, 200μm.

Fig.S3 *Drosophila* Trus and its paralog Zfrp8 share a core structural module that is evolutionarily conserved. **(A)** AlphaFold structure of *Drosophila melanogaster* Zfrp8 (Accession number: Q9W1A3) with domains PDCD2_N (green and light blue), PDCD2_C (magenta), β-strand (blue) that interacts with b-strand (light blue), and the MYND-type Zinc finger (red). **(B)** Alignment of the core module of *Drosophila* Trus (*Dm*Trus) to its paralog *Drosophila* Zfrp8 (*Dm*Zfrp8). **(C)** Alignment of the core module of *Drosophila* Trus (*Dm*Trus) with Zebrafish PDCD2L (*Danio rerio* PDCD2L). **(D)** Alignment of the core module of *Drosophila* Trus (*Dm*Trus) with yeast TSR4 (*Sc*TSR4). All structures presented are predicted by AlphaFold (https://alphafold.ebi.ac.uk/), and structural alignment was performed using PyMOL (https://pymol.org/2/).

Fig.S4 Trus protein is endogenously expressed in the central nervous system (CNS). CNS and disc tissues from *nub>TrusRNAi* 3rd instar wandering larvae were stained with affinity-purified anti-Trus antibody (green), Rhodamine-Phalloidin for F-actin (red), and DAPI for DNA (blue). Individual channels on the left and merged image on the right. Trus protein is expressed in brain lobes, the VNC, leg discs, eye-antenna discs, and the prothoracic gland.

Fig.S5 Trus localizes to the cytoplasm in cultured cells and *in vivo*. **(A)** *Drosophila* Trus localizes to the cytoplasm in S2 cells and shuttles between the nucleus and the cytoplasm in a CRM1-dependent manner. With no LB treatment (left panels), EGFP-Trus expressed in S2 cells localizes to the cytoplasm, and after treatment of the cells with Leptomycin B (middle and right panels),

bioRxiv preprint doi: https://doi.org/10.1101/2024.10.24.620039; this version posted October 26, 2024. The copyright holder for this preprint (which was not certified by peer review) is the author/funder, who has granted bioRxiv a license to display the preprint in perpetuity. It is made available under aCC-BY 4.0 International license.

an inhibitor of CRM1, EGFP-Trus accumulates in the nucleus and is depleted from the cytoplasm (LMB 15min, LMB 115min). Bar: 20mm. **(B)** *da>UAS-EGFP-Trus* is ubiquitously expressed in most tissues due to *daughterless* expression. EGFP-Trus (green) staining is shown to localize in the cytoplasm of the prothoracic grand (left panels), brain lobe and ventral nerve cord (second from left panels), and the wing disc (2 panels on right). Scale bar: 200mm. Magnified images of a wing pouch region are shown in the right column.

Fig.6S Enlarged lymph gland in trus1/trus1 larva. Lymph gland is marked with yellow bracket. Scale bar: 200µm.

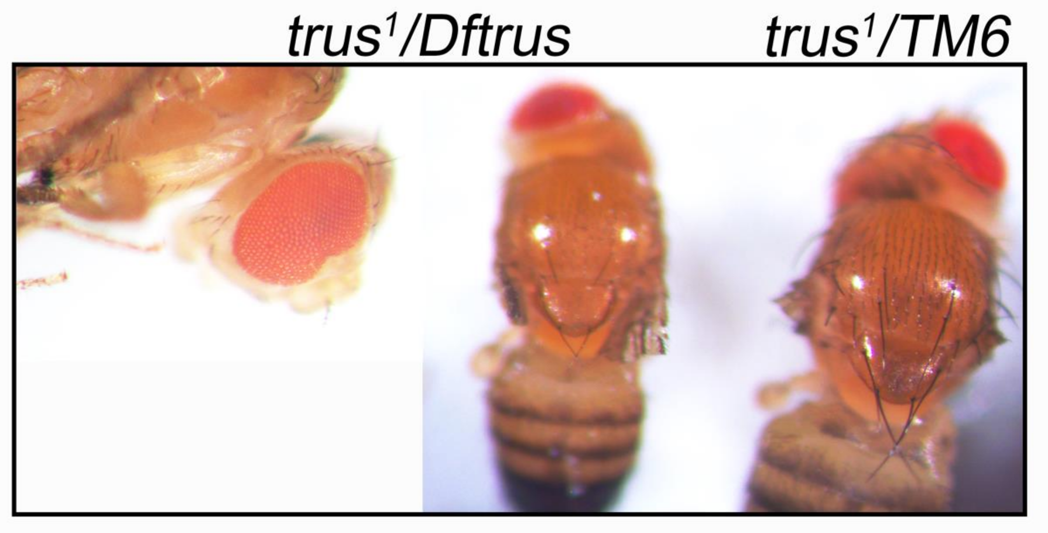

Fig.S1

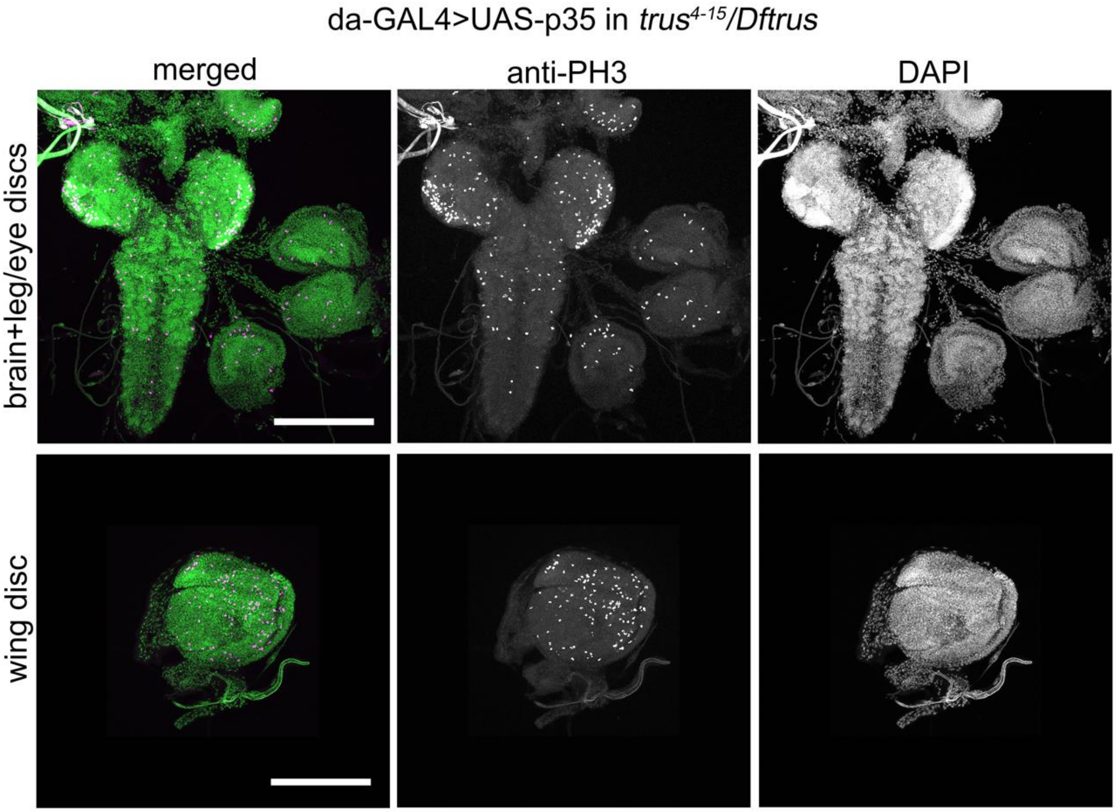
da-GAL4>UAS-p35 in $trus^{4\text{-}15}$/Dftrus
merged
anti-PH3
DAPI
brain+leg/eye discs
wing disc

Fig.S2

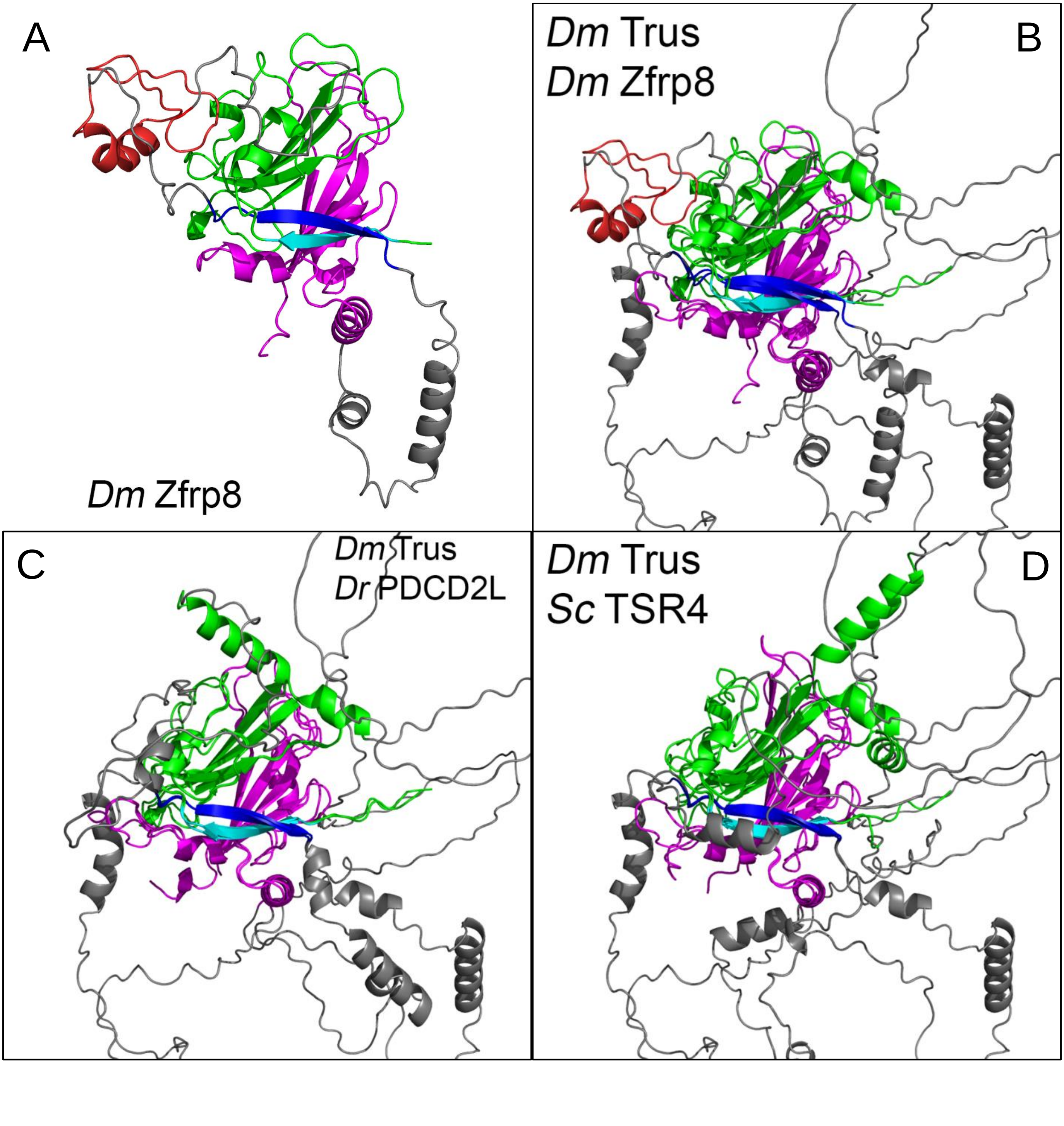
A
Dm Zfrp8
B
Dm Trus
Dm Zfrp8
C
Dm Trus
Dr PDCD2L
D
Dm Trus
Sc TSR4

Fig.S3

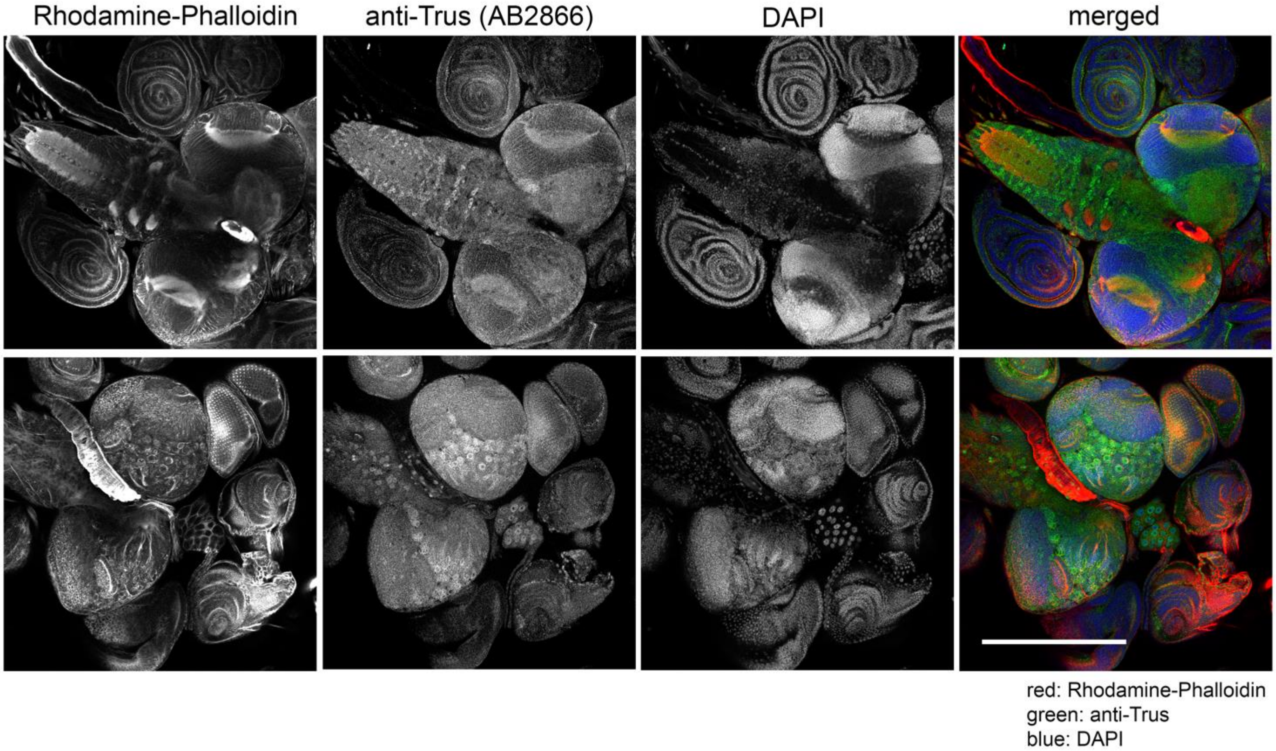
Rhodamine-Phalloidin
anti-Trus (AB2866)
DAPI
merged
red: Rhodamine-Phalloidin
green: anti-Trus
blue: DAPI

Fig.S4

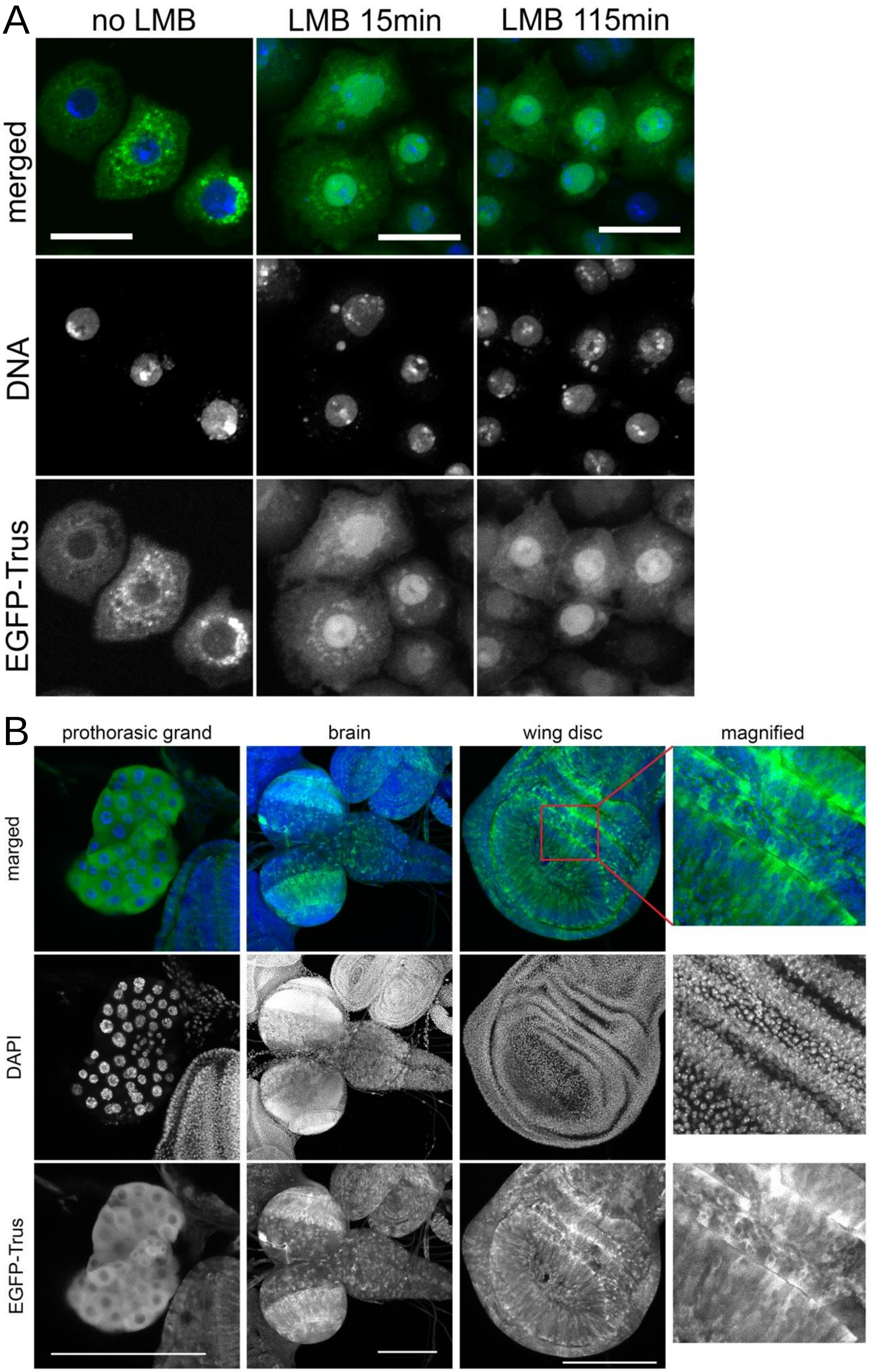
A
no LMB
LMB 15min
LMB 115min
merged
DNA
EGFP-Trus
B
prothorasic grand
brain
wing disc
magnified
marged
DAPI
EGFP-Trus

Fig.S5

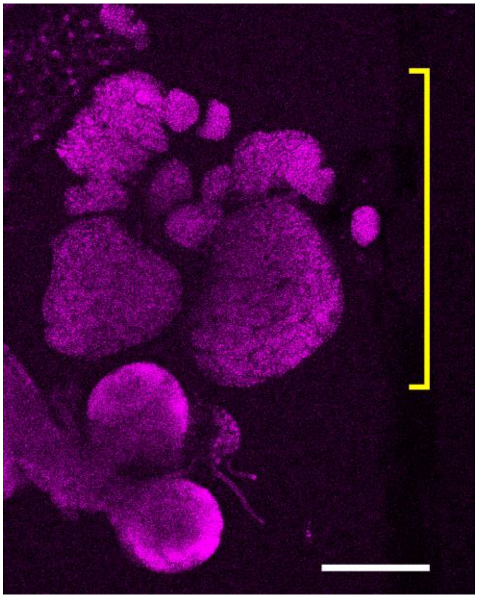

Fig.S6
